# Measuring Multisensory Integration in Clinical Settings: Comparing an Established Laboratory Method with a Novel Digital Health App

**DOI:** 10.3390/brainsci15060653

**Published:** 2025-06-17

**Authors:** Valerie Nunez, James Gordon, Mooyeon Oh-Park, Jessica Silvers, Tanya Verghese, Vance Zemon, Jeannette R. Mahoney

**Affiliations:** 1 Department of Neurology, Division of Cognitive and Sensorimotor Aging, Renaissance School of Medicine, Stony Brook University, Stony Brook, NY 11794, USA; valerie.nunez2@stonybrookmedicine.edu; 2 Department of Psychology, Hunter College, City University of New York, 695 Park Ave., New York, NY 10065, USA; jgordon@hunter.cuny.edu; 3 Burke Rehabilitation Hospital, 785 Mamaroneck Avenue, White Plains, NY 10605, USA; ohpark@burke.org; 4 Department of Rehabilitation Medicine, Albert Einstein College of Medicine, 150 East 210th Street, Bronx, NY 10467, USA; 5 School of Arts & Sciences, University of Rochester, 500 Joseph C. Wilson Boulevard, Rochester, NY 14627, USA; jsilve23@u.rochester.edu; 6 Ferkauf Graduate School of Psychology, Yeshiva University, 1165 Morris Park Ave., Bronx, NY 10461, USA; tanya.verghese@einsteinmed.edu (T.V.); vepman@aol.com (V.Z.); 7 Department of Neurology, Division of Cognitive & Motor Aging, Albert Einstein College of Medicine, Bronx, NY 10461, USA

**Keywords:** multisensory integration, visual–somatosensory integration (VSI), reaction time (RT), simple reaction time test, CatchU^®^

## Abstract

**Background/Objectives:** Recent research has correlated an inability to integrate sensory information with several adverse clinical outcomes, including slow gait, poor balance, and falls. For this reason, a digital health iPhone app (CatchU^®^ v3.1.2) has been strategically designed to bring the measurement of visual–somatosensory integration into clinical settings. The purpose of this study was to determine whether CatchU could reliably capture the phenomenon of multisensory integration compared to a validated piece of laboratory apparatus (“tristimulator”). **Methods**: Using both the established tristimulator and CatchU, 50 participants (76.5 ± 6.2 years of age, 60% female) completed a simple reaction time test in response to visual, somatosensory, and combined visual–somatosensory stimulation. A reaction time cumulative distribution frequency (CDF) curve was calculated for each stimulus condition, and together these were used to calculate the CDF difference function (the multisensory visual–somatosensory CDF minus a magnitude-limited sum of the unisensory visual and somatosensory CDFs). From this, the magnitude of visual–somatosensory integration (VSI) was obtained. **Results**: CatchU captured multisensory integration in both average reaction times and the CDF difference function. It also produced a similar magnitude of VSI and showed no systematic bias compared to the laboratory stimulator. Additionally, CatchU responses were significantly less variable than responses recorded using the tristimulator. **Conclusions**: Despite using different forms of stimulation and different methods to record responses, these results reveal that CatchU can be used to produce the same inferences as laboratory apparatus. This confirms the ability of CatchU to reliably capture VSI.

## 1. Introduction

Merging information from multiple senses improves our responses to the immediate environment. For example, by combining visual, somatosensory, and proprioceptive information, we automatically know where to place our feet safely as we walk down the street or on uneven ground. Beginning in the early 20th century [1], studies measuring simple reaction time (RT) have shown that responses to multisensory stimuli are typically faster than responses to either of the constituent unisensory stimuli, a response enhancement referred to as multisensory integration (MSI). Here we focus on visual–somatosensory integration (VSI), which has been observed in the higher-order association cortices of the prefrontal and parietal cortices, as well as between the primary visual and somatosensory cortices [2].

Older populations have varying levels of ability to integrate visual and somatosensory information, and poor ability may be linked to deficits in the neural pathways necessary for successful integration to occur [3]. This finding has led to the study of VSI processes and their relation to clinical outcomes in healthy and disease populations. While the relationship between VSI and cognitive, motor, and other clinical outcomes is still under-studied, recent investigations of VSI in older adults have correlated inability to integrate visual and somatosensory information with poor balance [3]; slow gait performance [4]; increased falls [3]; cognitive impairment (specifically attention issues) [5]; and, most recently, amyloid pathology associated with Alzheimer’s disease [6]. Collectively these findings highlight the clinical value of evaluating VSI in clinical settings. Indeed, a recent review proposed combining multisensory assessments with neuropsychological assessments for older adults “to improve the ecological validity of the neuropsychological assessment” [7]. To date, the above VSI studies have involved the use of a research laboratory instrument (the “tristimulator”—see Section 2.3.1) designed to optimize the presentation of stimuli and recording of RTs. The need for a method for the rapid evaluation of VSI in a clinical setting that can be administered remotely without assistance from providers or staff led to the development of a digital health app, CatchU^®^ (by one of the authors, J.R.M.) that emulates the tristimulator experiment on a smartphone. This app is easily accessible as it can be downloaded onto smartphones, obviating the need to purchase expensive and bulky equipment, design and code an experiment, and conduct independent data analyses. CatchU presents visual stimuli on the smartphone’s screen, uses built-in haptic actuators to produce vibrations that serve as somatosensory stimuli, and takes advantage of the touch-sensitive screen to detect responses. The CatchU app is user-friendly and automatically produces an analysis report upon completion of the test, using the same statistical analyses as the laboratory tristimulator. Given the inherent methodological differences between CatchU and the tristimulator (see Section 2.3), the aim of this study was to determine whether CatchU could reliably capture the phenomenon of MSI and evaluate the same psychophysical and behavioral responses as the laboratory apparatus.

Here, we used a within-subjects design to compare the ability of CatchU and tristimulator measurements to evaluate VSI. CatchU was able to determine VSI both in terms of faster multisensory than unisensory RTs, and in terms of the magnitude of VSI, a measure used in previous studies to quantify and categorize a person’s ability to integrate visual and somatosensory information [8]. The magnitude of VSI obtained using CatchU was not significantly different from that obtained using the tristimulator, and CatchU had a moderate agreement level with the tristimulator in classifying participants as integrators or non-integrators. CatchU also yielded much lower variability in its measurements. Together, these results indicate that, despite methodological differences between CatchU and the laboratory tristimulator, CatchU can reliably capture VSI.

## 2. Materials and Methods

### 2.1. Participants

The current experiment was conducted at Burke Rehabilitation Hospital (White Plains, NY, USA). Participants were recruited from the Burke Adult Fitness Center and outpatient therapy clinics in Westchester County, NY. All participants provided written informed consent to participate in this study, which is part of a larger investigator-initiated clinical trial that aims to demonstrate the acceptable-to-excellent predictive accuracy of CatchU to identify older adults at risk for falls [9]. The experiment was conducted in accordance with the principles embodied in the Declaration of Helsinki, and was approved by the Institutional Review Board of the Albert Einstein College of Medicine.

A total of 57 participants fulfilled the admission criteria of the parent clinical trial [9] and completed the RT experiment using both multisensory instruments between January 2023 and June 2024. However, two individuals were excluded because they scored below the revised recommended cut-off of 23 on the Montreal Cognitive Assessment (MoCA; [10,11]). Therefore, a total of 55 participants met the eligibility criteria for the study before considering test accuracy (see Section 3.1).

### 2.2. Clinical Evaluation

As part of the parent trial [9], participants underwent neuropsychological, gait, balance, and visual acuity testing. During a health screening interview, measures of global health were obtained from dichotomous (presence/absence) self-reported ratings of physician-diagnosed hypertension, arthritis, diabetes, depression, chronic obstructive pulmonary disease, myocardial infarction, angina, stroke, chronic heart failure, and Parkinson’s disease. One point was scored for the presence of each condition, and these ratings were combined into a global health score (GHS) ranging from 0 to 10. An assessment of neuropathy was also conducted using the Michigan Neuropathy Screening instrument [12].

### 2.3. Apparatus

**Figure 1:**
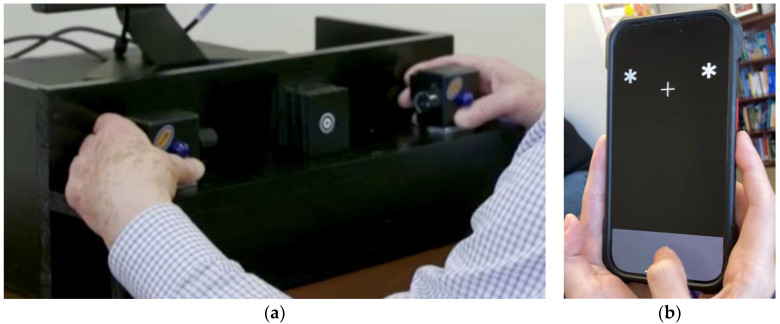
The two instruments: (**a**) The tristimulator: Here, a person is holding the control boxes on the tristimulator, with thumbs directly under the blue LEDs and fingers on the back of the boxes, with forefingers over the vibrating motors [8]. A bullseye fixation point is centered between the two control boxes. The foot pedal for responding is not visible, as it is under the table (**b**) CatchU app on an iPhone: The visual stimuli were two white asterisks on either side of a fixation cross. The gray area at the bottom of the screen is where the participant tapped to respond to each stimulus.

The two multisensory experimental instruments were a desktop sensory stimulator (“tristimulator”) and “CatchU”—a digital health application presented on an iPhone (see Figure 1). Each will be described in turn.

#### 2.3.1. Tristimulator

The visual and somatosensory stimuli were provided by a custom-built stimulus generator (Zenometrics LLC; Peekskill, NY, USA). The third stimulation modality of the tristimulator—sound—was not included in the current experimental design. The tristimulator has been described in detail elsewhere [3]; however, for completeness some details will be provided here. It consisted of two control boxes with vibrators on the back and blue light-emitting diodes (LEDs) on the front (see Figure 1a). Visual stimuli were produced by the blue LEDs (diameter 15.88 mm) on the front of each control box. Somatosensory stimuli were produced by a motor with a 0.8 G vibration amplitude on the back of each box. A third box was centered equidistant between the two control boxes, with a small bullseye on the front serving as a fixation point. At a viewing distance of 57 cm, the centers of the LEDs were located 18° of visual angle each side of the fixation point, and the fixation circle subtended a visual angle of 0.4°. Responses to visual and/or somatosensory stimuli were recorded by foot pedal presses from the right foot. Participants wore headphones that emitted continuous white noise to mask any sound from the vibration motors, thereby ensuring that participants only responded to the tactile sensation of the vibration, rather than to its sound. The presentation of stimuli and recording of responses were controlled using E-Prime^®^ 2.0 software.

#### 2.3.2. CatchU^®^

CatchU (a JET Worldwide Enterprises Inc. product; Stony Point, NY, USA) is a mobile multisensory RT app available on iPhone and Android devices, developed by J.R.M. to identify older adults at risk of falling [13]. In this study, all participants completed the CatchU test (version 3.1.2) on an Apple iPhone 7 (height 13.8 cm, width 6.7 cm, operating system iOS 15.7). Throughout the duration of a session, the app displayed a plain black background with a gray response section at the bottom (see Figure 1b). Responses to visual and/or somatosensory stimuli were provided by tapping on the response area. A white fixation cross (centered) was displayed 3.8 cm from the top of the iPhone throughout the duration of the experiment. The visual stimulus consisted of two large white asterisks (0.8 cm in diameter) that would simultaneously appear on both sides of the fixation cross, just above it (see Figure 1b). As the distance at which the phone was held was not specified, there would have been some natural variation in the distance of the screen from the participants’ eyes, resulting in variation in the visual angle subtended by the stimuli. The somatosensory stimulus consisted of tactile vibrations (as may happen when a cellphone rings on vibration mode) that were triggered through Apple’s hardware (Taptic Engine). Any sound of the vibration was masked by continuous white noise played out loud by CatchU during the experiment. The presentation of the various stimuli and recording of all RT responses were controlled automatically through the CatchU app.

### 2.4. Experimental Procedure

The tristimulator had two control boxes mounted on its front, and participants were instructed to hold these with each hand, with thumbs directly under the blue LED on the front (so as not to block the view of the visual stimulus) and forefingers over the vibrating motor on the back of each box (as in Figure 1a). Participants were instructed to respond to each stimulus (regardless of type) by pushing a foot pedal positioned under their right foot.

For CatchU, participants were asked to cradle the bottom half of the iPhone firmly between both hands (with the iPhone sitting in the palms of both hands, fingers supporting the back of the phone, and maximum contact over the bottom portion of the iPhone), enabling the participant to feel vibrations without blocking their view of the visual stimuli, while still having their thumbs free to tap the response area (as in Figure 1b). Participants were instructed to respond to each stimulus by tapping the response area of the screen with the thumb of their dominant hand.

Other than the difference in stimulus presentation modes given the inherent differences in technology (phone vs. computer and tristimulator) and responses (dominant thumb vs. right foot), the rest of the experimental design was the same across instruments.

Participants were seated comfortably in a well-lit room. A single trial consisted of the presentation of a visual, somatosensory, or combined visual–somatosensory stimulus, or no stimulus at all, for a period of 100 ms. Participants were given a maximum of 2000 ms from stimulus onset to respond. If a response was made, the RT (time from stimulus onset to response) was recorded. If no response was made within the 2000 ms response window, a default value representing no response was recorded. For subsequent stimuli, the response–stimulus interval (or, in the case of no response, the time from the end of the response window to the onset of the next stimulus) was randomized between 1000 and 3000 ms to prevent anticipation of the next stimulus.

Both instruments included practice sessions of five trials each of the unisensory (visual or somatosensory) and multisensory (visual–somatosensory) stimuli. These were presented in pseudo-random order (limited to present no more than three identical stimuli in a row). Participants were asked to focus on the fixation point and to respond as quickly as possible once they felt and/or saw any stimulus. If unable to follow the directions or respond to the practice trials, a participant was given the chance to repeat the practice trial once more before the test was deemed invalid and the experiment was ended.

The actual experiment consisted of three blocks of 60 trials. Each block contained 15 trials of each stimulus type (visual, somatosensory, visual–somatosensory, or no stimulus) presented in pseudo-randomized order, as during the practice session. The three experimental blocks, consisting of a total of 180 trials, were separated by a 20 s rest period to reduce fatigue and enhance attention. The total test time was, on average, 10 min across both instruments (ranging from 9 to 12 min depending on individuals’ reaction times and number of misses).

### 2.5. Preprocessing of Data

CatchU automatically conducts a complete analysis of RT data up to the point of producing a report for providers, including graphs and feedback for their consideration (adapted from CDC’s STEADI initiative [14]) based on the participant’s individual responses and multisensory performance. However, to ensure that both instruments were compared using identical analyses, the raw RT data from CatchU and ePrime (for the tristimulator) were imported into Matlab, filtered (see below), and sorted in order of increasing RT; then, they were used to populate specific data fields in Excel files containing ready-made formulae and graphs for the analysis. That way, the analyses for both instruments started with raw RT data that was subjected to the same data analysis pipeline. Following the protocol outlined by Mahoney & Verghese [8], for each participant, the RTs across the three experimental blocks were combined for each stimulus condition and non-responses were given an RT value set to infinity. The remaining data were then filtered in two ways: first, RTs less than 100 ms were set to infinity (assuming that such fast responses were either late responses to the prior stimulus, anticipation of the current stimulus, or simply not related to the stimuli at all (see, e.g., [15,16])). Then, for each stimulus condition, RTs (excluding those set to infinity) outside the range of the mean RT ± 2.5 *SD* were also set to infinity to prevent outliers from contributing to the cumulative RT distribution function. Lastly, consistent with most recent work including participants with mild cognitive impairment [6], a response accuracy of less than 60% on any one condition (where *n* = 45 trials per condition) was considered invalid, and participants with invalid data were excluded.

### 2.6. Reaction Time Probability Distributions and the CDF Difference for the Race Model Inequality

For each condition, sorted RT data were grouped into percentile bins (in 5% increments) from fastest RT (0.00 percentile) to slowest RT (1.00 percentile) and were used to calculate the cumulative distribution functions (CDFs), indicating the probability of a response occurring by the time of any given percentile bin.

In the context of Miller’s race model inequality [17], which tests the assumption of separate activation vs. coactivation during bimodal RT tasks, when the CDF for the combined visual–somatosensory stimulus is larger than the sum of the CDFs for the two corresponding unisensory stimuli, the race model is violated, and VSI has occurred [18,19,20]. Colonius & Diederich [21] modified this inequality by limiting the sum of the CDFs for the unisensory visual and somatosensory stimuli to a maximum value of 1. Then they plotted the CDF for the combined stimulus minus the modified sum of the two unisensory CDFs (the “CDF difference”) and showed that the race model was rejected (in favor of coactivation, i.e., MSI) when the CDF difference values were above zero. Following the method of Colonius & Diederich [21] the CDF difference value was calculated and plotted for each participant using both the tristimulator and CatchU RTs. Positive values at any given latency (i.e., percentile bin) are indicative of successful MSI. The area under the CDF difference curve (AUC) up to and including the bin corresponding to the 10th percentile of the RT range represents the magnitude of VSI (see [8] for detailed specifications). A positive magnitude of VSI indicates that a person was able to successfully integrate visual and somatosensory information (i.e., “integrator”), whereas a negative magnitude of VSI indicates that a person was unable to benefit from receiving concurrent visual and somatosensory information (i.e., “non-integrator”) [4]. The magnitude of VSI was calculated for each participant for each instrument.

### 2.7. Statistical Analyses

Descriptive statistics (*M* and *SD*) were calculated for RTs, and 2 × 3 (instrument × stimulus condition) repeated measures ANOVAs were conducted. All statistical data analyses were run using JASP statistical software v0.19.3 [22].

## 3. Results

### 3.1. Demographic Information

After excluding participants who produced invalid data (less than 60% accuracy in any of the three sensory modalities), a total of 50 participants (20 males, mean age = 76.8 years (*SD* = 5.4); 30 females, mean age = 76.3 (*SD* = 6.7)) had data that could be compared directly across the two instruments. Demographic information is provided in Table 1.

### 3.2. Reaction Times

**Figure 2:**
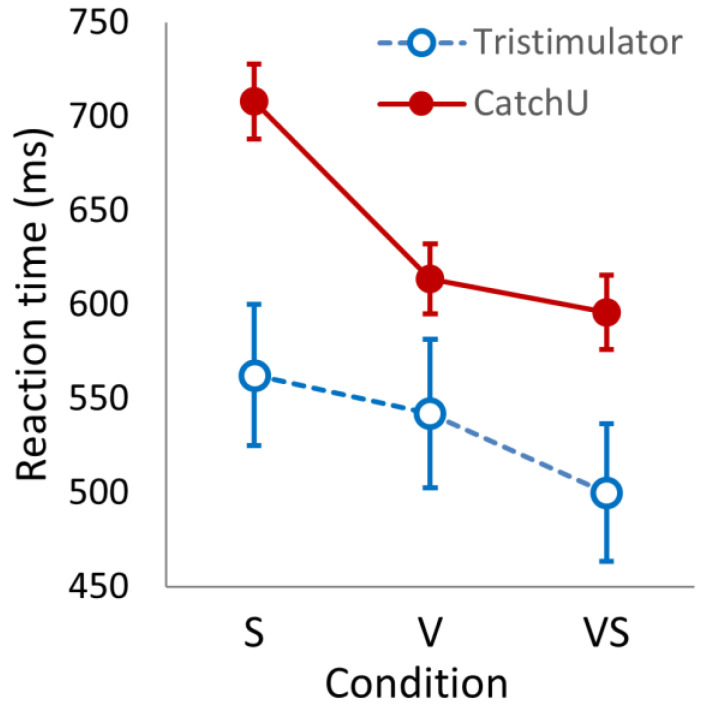
The reaction times (in ms) for the three stimulus conditions (somatosensory, S; visual, V; and visual–somatosensory, VS) recorded with CatchU (filled red circles) and with the tristimulator (open blue circles). The error bars represent ±1 *SEM*.

The mean RT per condition was grand-averaged over participants for each instrument—the results are provided in Table 2. Looking at the descriptive statistics of Table 2, for both instruments, the somatosensory mean RTs are slower than the visual responses, which, in turn, are slower than the visual–somatosensory responses. In addition, the mean responses measured with CatchU are slower than the tristimulator responses. A 2 × 3 ANOVA (instrument × condition) indicated significant main effects of the instrument (*F*(1, 49) = 9.184, *p* = 0.004) and condition (*F*(2, 98) = 21.629, *p* < 0.001) as well as a significant interaction (*F*(2, 98) = 120.289, *p* < 0.001)—see Figure 2.

From Table 2, one can also see that the coefficients of variation (*CV*s) and intra-individual variability (*IIV*) are much larger for tristimulator responses compared to CatchU—indeed, for each condition, the tristimulator *CV* is more than double the *CV* for CatchU. A repeated measures 2 × 3 (instrument × condition) ANOVA of *IIV* showed a significant main effect of the instrument (*F*(1, 49) = 58.054, *p* < 0.001) and a large effect size (η^2^ = 0.420).

### 3.3. CDF Difference Curves

**Figure 3:**
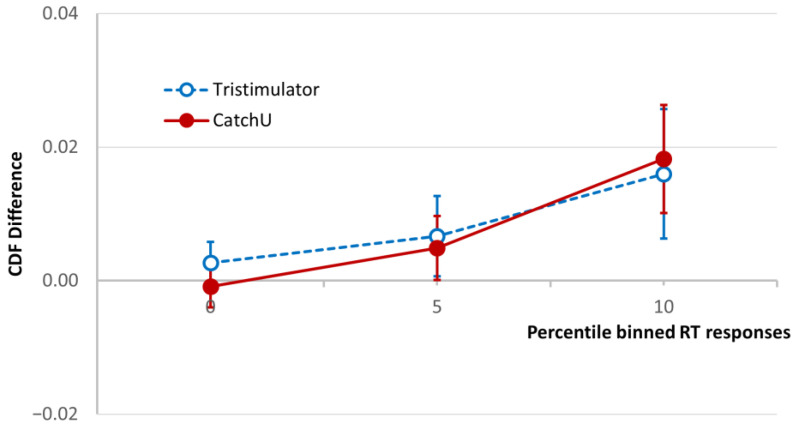
Test of the race model. The CDF difference averaged for the study cohort using CatchU (solid red curve) and the tristimulator (dashed blue curve) up to 10th-percentile RT bin. The CDF difference is the difference between the CDF of the visual–somatosensory responses and the modified sum of the unisensory visual and somatosensory CDFs ([21], see Section 2.6). The error bars represent ±1 *SEM*. The area under the CDF difference curve (AUC) up to and including the 10th-percentile bin is used in the standard protocol [8] to determine whether a participant is a visual–somatosensory integrator.

The cumulative distribution functions for the three conditions, visual, somatosensory, and visual–somatosensory, were used to calculate the CDF difference of Colonius & Diederich [21], and the grand-averaged CDF difference curves for both instruments are plotted in Figure 3 for the fastest 10th percentile of the RT range [4,8]. As described above (Section 2.6), if the AUC in this RT range (magnitude of VSI) is positive, the participant is considered a visual–somatosensory integrator, and if it is negative they are considered a non-integrator.

Looking at Figure 3, one can see for the plotted RT percentile range that the grand-averaged CDF difference is similar for both instruments, and both demonstrate overall MSI in the sample. The total AUC in the same range (magnitude of VSI) was calculated for both instruments for each participant, and a paired samples *t* test indicated no significant difference in AUC between the two instruments (*p* = 0.837)—which is also evidenced by the overlapping SEM bars.

### 3.4. Bland–Altman Analysis

**Figure 4:**
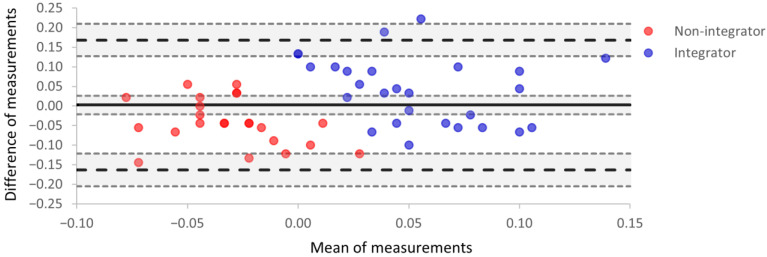
A Bland–Altman plot indicating the level of agreement between parameters obtained using tristimulator and CatchU. The x-axis is the mean magnitude of VSI for the two instruments, the y-axis is the difference between the two magnitudes, and each participant is represented by one point on the plot. The mean difference in measurements represents the systematic bias (*B*) between the two instruments and is indicated by the thick solid black line. The upper and lower limits of agreement delineate a range given by *B* ±1.96 *SD* and are represented as black dashed lines. Around each of these three lines, the shaded areas are the 95% confidence intervals. The red points represent participants who were not multisensory integrators according to tristimulator measurements, i.e., the magnitude of VSI was negative for those participants. The blue points represent integrators based on the tristimulator measurements. Points with a more saturated color represent overlapping data for two participants.

For each participant, the magnitude of VSI for each instrument was used in a Bland–Altman plot (Figure 4). In Figure 4, agreement between the two instruments is represented by one point on the plot for each participant. The x-axis is the mean magnitude of VSI for the two instruments; the y-axis is the difference between the two magnitudes (tristimulator–CatchU).

The mean difference, *B*, in VSI magnitudes is the estimated bias between the two instruments, and the bias confidence interval (the shaded area around the thick black line in Figure 4) includes zero; therefore, there is no significant systematic bias between the two instruments. The values of bias and corresponding limits of agreement from Figure 4 are presented in Table 3. The range of limits of agreement is somewhat larger than the range of mean measurements. However, only two individual points lie outside the upper limit of agreement.

In Figure 4, red points represent participants who were defined as visual–somatosensory non-integrators (i.e., the magnitude of VSI was negative for those participants), and blue points represent visual–somatosensory integrators; the designations here are based on tristimulator performance. The red non-integrator points mostly have negative abscissa values, while the blue multisensory integrator points have positive abscissa values. While most integrators had a mean of measurements greater than zero, there were three *non-integrators* with values greater than zero (red points with positive x values). This reveals non-integrator status on the tristimulator but integrator status on CatchU for these individuals. This serves as a visual demonstration that the two instruments may sometimes provide different conclusions (non-integrator vs. integrator) for the same participant when using a cut-off of AUC > 0.00 to define integrator status.

Comparing how well the two instruments agree on categorizing a participant as an integrator or non-integrator, the contingency table (Table 4) demonstrates a 70% level of agreement, which is considered moderate.

## 4. Discussion

The main objective of this study was to determine whether a digital health smartphone app could be used to replace the bulky and prohibitively expensive specialized research instrument frequently used to evaluate VSI in the laboratory. Here we compare the performance of CatchU to that of the tristimulator using a few different measures.

### 4.1. Reaction Times

The tristimulator and CatchU produced differences in the RTs and coefficients of variation. CatchU recorded slower response times than the tristimulator across all stimulus conditions. Given our knowledge of response rates from the hand and foot, this was unexpected. Previous research has shown that hand responses are faster than foot responses [23,24,25]. In addition, the dominant hand/foot responds faster than the non-dominant hand/foot [23], and the thumb tends to exhibit the fastest responses of the digits [26]. Therefore, we expected that a response by the thumb of the dominant hand would be faster than a response by the right foot (regardless of foot dominance). However, this was not the case. This leads us to conclude that slower RTs captured from CatchU were most likely due to the stimuli being less salient than those of the tristimulator.

There are a few potential explanations for the tristimulator visual RTs being faster than the CatchU RTs. First, cortical response times are faster for increased retinal illumination [27], and the tristimulator lights did subtend a larger visual angle than the white asterisks of CatchU. In addition, the two stimuli may have elicited responses from different classes of photoreceptors and neurons in the visual pathway due to different subtended visual angles and stimulus types. For example, diffuse colored stimuli (such as the blue lights of the tristimulator) stimulate specific cortical neurons that respond faster than the neurons that respond to patterned stimuli (such as the white asterisks of CatchU) [28]. Consequently, a future version of CatchU could have one larger central stimulus on the screen to speed up recorded visual response times.

Concerning somatosensory responses, there are some possible reasons why the CatchU somatosensory stimuli may have been less salient than those of the tristimulator. The tristimulator’s vibration motors were directly against the participants’ forefingers and had been set to a salient amplitude of vibration. The iPhone’s vibrating actuator (Apple’s Taptic engine) is set at a fixed vibration amplitude that could not be further enhanced (a restriction of the device). Inside the iPhone 7 (the phone used for all participants enrolled in this study) the vibrating actuator is along the bottom right side of the phone, covering the bottom quarter of the screen. Looking at Figure 1b, one can see that the participants’ fingers would not have been touching the part of the phone with the strongest vibration. As the actuator’s direct contact was mostly against the palm instead of the fingertips, this would also have reduced the sensitivity to the vibration (see, for example [29]), thereby potentially delaying the response time. This aspect of using CatchU can be improved by providing an instructional video that demonstrates a standardized way to hold the phone to optimize the salience of the somatosensory stimuli.

For the purposes of this study, however, the relevant question was whether CatchU had the capability to establish and evaluate the effect of multisensory integration. In Table 2 and Figure 3, the grand-averaged response time for the multisensory stimuli is faster than for each of the unisensory modes of stimulation as recorded using CatchU. Therefore, from the RTs alone, CatchU is clearly able to ascertain this phenomenon.

An important finding was that the CatchU responses are significantly less variable than the tristimulator responses, both within and between participants. This indicates that results with CatchU should be more reliable and consistent than with the tristimulator. This is a promising result for the potential use of CatchU in clinical settings.

### 4.2. CDF Difference Curves

Having established faster RTs for visual–somatosensory stimuli compared to unisensory stimuli using CatchU, we needed to determine whether VSI effects were evident in the first 10% of RTs for CatchU data. As shown in Figure 3, the CatchU CDF difference curve was similar to that of the tristimulator up to the 10th-percentile RT bin. This corresponds to the range over which the AUC is calculated historically to determine whether a participant is a visual–somatosensory integrator; in this range the AUC was not significantly different between the two instruments. This is noteworthy given differences in both stimulation delivery and response recording across the two instruments.

One should also note that the 0–10th-percentile RT range was originally determined using the same tristimulator with a large dataset from a study conducted in the Bronx, NY (the Central Control of Mobility in Aging (CCMA) study [4]). Here, the cohort of participants was completely different both demographically and clinically, as can be seen by comparing Table 1 with the demographic Table 1 of the Bronx study [4]. This makes the level of agreement between the instruments in the CDF over this range even more remarkable and indicates potential generalizability across different participant cohorts.

### 4.3. Bland–Altman Analysis

The Bland–Altman analysis indicated no significant bias between the data for CatchU and the tristimulator. Concerning the observation that the limits of agreement were relatively large compared to the range of the means, the variance in the difference scores was increased primarily by the greater variability in the tristimulator measurements.

Comparing the classification of multisensory integrators and non-integrators determined using the tristimulator and CatchU, the two instruments had an agreement level of 70%. This indicates adequate agreement; however, it is unclear which of the two instruments better identifies integrators. Considerably less variability was inherent in the CatchU measurements, likely due to the different experimental conditions (responses from the finger vs. foot, different hardware configurations, etc.). We speculate, therefore, that greater true integrator classification might be obtained with CatchU. We anticipate that this question will be answered, in terms of classifying risk of falls, at the conclusion of the larger investigator-initiated clinical trial [9].

### 4.4. Other Instrumental Considerations

The tristimulator malfunctioned during the last part of data collection for this study, highlighting one major advantage of using CatchU over the tristimulator, as iPhone actuators are expected to be considerably more reliable. More importantly, the main advantage of using a smartphone app is that it is easily accessible, much cheaper than a piece of research equipment, and is less cumbersome, so it is not restricted to use in the laboratory. The CatchU app is also available on Android phones, so this makes the measurement of VSI even more accessible. Although the app may be used on different phones with different screens and vibrating actuators, it will nevertheless provide the same unisensory and multisensory stimuli within each experiment, affording accurate capture of the multisensory phenomenon. As we have demonstrated, even when the phone is held in less-than-optimal ways, it is possible to measure VSI so long as the physical conditions are kept constant during the entire experiment.

### 4.5. Future Work

The comparisons here used the same zero AUC cut-off and percentile of the RT range (as defined in the published protocol [8]) to determine integrator/non-integrator status for the tristimulator and CatchU. However, these may not be consistent across both instruments—future directions include the preparation of normative data by age and sex to determine proper cut-offs for integration vs. non-integration. From this we should be able to determine if we can further enhance the accuracy of CatchU to determine whether someone is an integrator.

The Mahoney laboratory is currently conducting a study of VSI as a potential biomarker of Alzheimer’s disease [30]. The study uses a stimulator that records responses from the fingertips by having the user press the same buttons that deliver the vibrations. In addition, multisensory RT data are recorded during fMRI sessions, using tiny pneumatic balloons that inflate to produce somatosensory sensations, and MRI-safe goggles that display visual stimuli. MSI is also being evaluated for the same participants using CatchU. A comparison of performance across these three different instruments, within the same population, should aid in the determination of the degree of generalizability of the technique across various platforms that measure VSI.

The data analysis performed here was based on the work of Miller [17], Colonius & Diederich [21], Gondan & Minakata [20], and others. It involves calculating the CDF difference for each participant. However, there are several different models of multisensory integration (for a review see [31]) as well as different recommendations relating to filtering (e.g., [32,33]), treating missing values (e.g., [33,34]), fast guesses (e.g., [35]), and other treatments of RT data (see, e.g., [15,36,37]) that could be considered in addition to the previously described analysis [8]. For example, some studies have used the shape of the RT distribution to infer information about attention [38,39]. Such analyses could enhance or supplement the information obtained using the AUC of the CDF difference curve and will be examined in subsequent studies.

## 5. Conclusions

CatchU captures the phenomenon of MSI in both average RT and in the CDF difference curve. It also produces a similar magnitude of VSI and shows no systematic bias compared to the laboratory tristimulator. Additionally, CatchU responses are significantly less variable than responses recorded using the laboratory instrument. Therefore, despite using different forms of stimulation and different methods to measure responses, CatchU can be used to produce the same inferences as the laboratory tristimulator. This highlights the strength of the strategic design of the MSI test, whereby the unisensory stimuli effectively serve as control stimuli against which the multisensory condition is compared. To summarize, the results from this study reveal that CatchU can reliably ascertain and measure visual–somatosensory integration.

## Figures and Tables

**Table 1:** Demographic and clinical characteristics of the sample.

Variable	Value
Number of participants, N	50
% Female	60.0
Age (y)	76.5 (6.2), range 65–89
% Caucasian	94.0
Education (y)	17.4 (3.8), range 5–28
% Visual impairment	54.0
% Neuropathy	12.0
GHS ^#^ (0–10)	2.3 (1.4), range 0–6
MoCA score	27.1 (1.6), range 24–30

^#^ GHS = global health score; MoCA = Montreal Cognitive Assessment test; y = years. Values are presented as *M* (*SD*) and range for continuous variables and % for dichotomous variables.

**Table 2:** Grand-averaged RTs (in ms) plus measures of variability between and within participants using the different instruments for the three stimulus conditions.

Instrument	Condition	Mean	*SD*	*CV*	*IIV*
CatchU	S	708.0	139.9	0.198	0.133
	V	613.7	131.6	0.214	0.136
	VS	595.9	139.7	0.234	0.121
Tristimulator	S	562.5	266.0	0.473	0.207
	V	542.1	280.1	0.517	0.226
	VS	500.0	258.1	0.516	0.209

Note: The different stimulus conditions are presented as follows: S = somatosensory; V = visual; and VS = visual–somatosensory. *CV*, the coefficient of variation, is *SD* divided by the mean, and indicates the variability between participants for the various conditions. *IIV*, the intra-individual variability, is the grand-averaged coefficient of variation for each condition, i.e., indicates the average variability in RTs within participants and accounts for a situation where a participant may not have responded to all 45 stimulus presentations of a given condition.

**Table 3:** Bias, limits of agreement, and their 95% confidence intervals (CI) for the Bland–Altman test of Figure 4.

Bias and Limits of Agreement	Point Value	Lower 95% CI	Upper 95% CI
Bias (*B*)	0.002	−0.022	0.026
Upper limit of agreement (*B* + 1.96 *SD*)	0.168	0.127	0.209
Lower limit of agreement (*B* − 1.96 *SD*)	−0.163	−0.205	−0.122

**Table 4:** Contingency table indicating level of agreement between CatchU and tristimulator in categorizing integrators and non-integrators.

		Tristimulator	
		Non-Integrator	Integrator
**CatchU**	**Non-integrator**	16	8
	**Integrator**	7	19

Note: An integrator has a positive AUC of the CDF difference curve in the region up to and including the 10th-percentile RT bin.

## Data Availability

The datasets presented in this article are not readily available because the data are part of an ongoing clinical trial. When it is completed, requests to access these data should be directed to J.R.M.

## Abbreviations

The following abbreviations are used in this manuscript:

RT	Reaction time
MSI	Multisensory integration
VSI	Visual–somatosensory integration
CDF	Cumulative distribution function
AUC	Area under the curve
CV	Coefficient of variation
IIV	Intra-individual variability

## References

[1] Todd J.W., Woodworth R.S. (1912). Reaction to multiple stimuli. Archives of Psychology.

[2] Murray M.M., Thelen A., Thut G., Romei V., Martuzzi R., Matusz P.J. (2016). The multisensory function of the human primary visual cortex. Neuropsychologia.

[3] Mahoney J.R., Cotton K., Verghese J. (2019). Multisensory Integration Predicts Balance and Falls in Older Adults. J. Gerontol. A Biol. Sci. Med. Sci..

[4] Mahoney J.R., Verghese J. (2018). Visual-Somatosensory Integration and Quantitative Gait Performance in Aging. Front. Aging Neurosci..

[5] Mahoney J.R., Verghese J. (2020). Does Cognitive Impairment Influence Visual-Somatosensory Integration and Mobility in Older Adults?. J. Gerontol. A Biol. Sci. Med. Sci..

[6] Mahoney J.R., Ayers E., Verghese J. (2025). Visual-somatosensory integration as a novel behavioral marker of amyloid pathology. Alzheimers Dement..

[7] Pinto J.O., Bastos V.D.M.B., Bruno P., Andreia G., Barbosa F. (2021). Narrative review of the multisensory integration tasks used with older adults: Inclusion of multisensory integration tasks into neuropsychological assessment. Expert. Rev. Neurother..

[8] Mahoney J.R., Verghese J. (2019). Using the Race Model Inequality to Quantify Behavioral Multisensory Integration Effects. J. Vis. Exp..

[9] Mahoney J.R. CatchU: A Quantitative Multisensory Falls-Assessment Randomized Clinical Trial. ClinicalTrials.gov identifier: NCT05544760. NCT05544760.

[10] Carson N., Leach L., Murphy K.J. (2018). A re-examination of Montreal Cognitive Assessment (MoCA) cutoff scores. Int. J. Geriatr. Psychiatry.

[11] Thomann A.E., Berres M., Goettel N., Steiner L.A., Monsch A.U. (2020). Enhanced diagnostic accuracy for neurocognitive disorders: A revised cut-off approach for the Montreal Cognitive Assessment. Alzheimer’s Res. Therapy.

[12] Feldman E.L., Stevens M.J., Thomas P.K., Brown M.B., Canal N., Greene D.A. (1994). A Practical Two-Step Quantitative Clinical and Electrophysiological Assessment for the Diagnosis and Staging of Diabetic Neuropathy. Diabetes Care.

[13] Mahoney J.R., George C.J., Verghese J. (2022). Introducing CatchU(TM): A Novel Multisensory Tool for Assessing Patients’ Risk of Falling. J. Percept. Imaging.

[14] Algorithm for Fall Risk Screening, Assessment, and Intervention. https://www.cdc.gov/steadi/media/pdfs/STEADI-Algorithm-508.pdf.

[15] Whelan R. (2008). Effective analysis of reaction time data. Psychol. Rec..

[16] Luce R.D. (1991). Response Times: Their Role in Inferring Elementary Mental Organization.

[17] Miller J. (1982). Divided attention: Evidence for coactivation with redundant signals. Cogn. Psychol..

[18] Miller J., Ulrich R. (2003). Simple reaction time and statistical facilitation: A parallel grains model. Cogn. Psychol..

[19] Miller J. (1986). Timecourse of coactivation in bimodal divided attention. Percept. Psychophys..

[20] Gondan M., Minakata K. (2016). A tutorial on testing the race model inequality. Atten. Percept. Psychophys..

[21] Colonius H., Diederich A. (2006). The race model inequality: Interpreting a geometric measure of the amount of violation. Psychol. Rev..

[22] JASP Team (2025). JASP.

[23] Montés-Micó R., Bueno I., Candel J., Pons A.M. (2000). Eye-hand and eye-foot visual reaction times of young soccer players. Optometry.

[24] Hoffmann E.R. (1991). A comparison of hand and foot movement times. Ergonomics.

[25] Thomas P.K., Sears T.A., Gilliatt R.W. (1959). The Range of Conduction Velocity in Normal Motor Nerve Fibres to the Small Muscles of the Hand and Foot. J. Neurol. Neurosur Psychiatry.

[26] Wilimzig C., Ragert P., Dinse H.R. (2012). Cortical topography of intracortical inhibition influences the speed of decision making. Proc. Natl. Acad. Sci. USA.

[27] Mazade R., Jin J., Rahimi-Nasrabadi H., Najafian S., Pons C., Alonso J.M. (2022). Cortical mechanisms of visual brightness. Cell Rep..

[28] Nunez V., Gordon J., Shapley R. (2022). Signals from Single-Opponent Cortical Cells in the Human cVEP. J. Neurosci..

[29] Johansson R.S., Vallbo A.B. (1979). Tactile sensibility in the human hand: Relative and absolute densities of four types of mechanoreceptive units in glabrous skin. J. Physiol..

[30] Mahoney J.R., Blumen H.M., De Sanctis P., Fleysher R., Frankini C., Hoang A., Hoptman M.J., Jin R., Lipton M., Nunez V. (2023). Visual-somatosensory integration (VSI) as a novel marker of Alzheimer’s disease: A comprehensive overview of the VSI study. Front. Aging Neurosci..

[31] Colonius H., Diederich A. (2020). Formal models and quantitative measures of multisensory integration: A selective overview. Eur. J. Neurosci..

[32] Berger A., Kiefer M. (2021). Comparison of Different Response Time Outlier Exclusion Methods: A Simulation Study. Front. Psychol..

[33] Lachaud C.M., Renaud O. (2011). A tutorial for analyzing human reaction times: How to filter data, manage missing values, and choose a statistical model. Appl. Psycholinguist..

[34] Frey A., Spoden C., Goldhammer F., Wenzel S.F.C. (2018). Response time-based treatment of omitted responses in computer-based testing. Behaviormetrika.

[35] Miller J., Lopes A. (1991). Bias produced by fast guessing in distribution-based tests of race models. Percept. Psychophys..

[36] Rousselet G.A., Wilcox R.R. (2020). Reaction Times and other Skewed Distributions. Meta-Psychology.

[37] Van Zandt T. (2000). How to fit a response time distribution. Psychon. Bull. Rev..

[38] Hervey A.S., Epstein J.N., Curry J.F., Tonev S., Eugene Arnold L., Keith Conners C., Hinshaw S.P., Swanson J.M., Hechtman L. (2006). Reaction time distribution analysis of neuropsychological performance in an ADHD sample. Child. Neuropsychol..

[39] Yamashita A., Rothlein D., Kucyi A., Valera E.M., Germine L., Wilmer J., DeGutis J., Esterman M. (2021). Variable rather than extreme slow reaction times distinguish brain states during sustained attention. Sci. Rep..

